# The Human Mycobiome: Composition, Immune Interactions, and Impact on Disease

**DOI:** 10.3390/ijms26157281

**Published:** 2025-07-28

**Authors:** Laura Carrillo-Serradell, Jade Liu-Tindall, Violeta Planells-Romeo, Lucía Aragón-Serrano, Marcos Isamat, Toni Gabaldón, Francisco Lozano, María Velasco-de Andrés

**Affiliations:** 1 Immunoreceptors of the Innate and Adaptive Systems, Institut d’Investigacions Biomèdiques August Pi I Sunyer (FCR-IDIBAPS), 08036 Barcelona, Spain; lcarrillo@recerca.clinic.cat (L.C.-S.); planellsi@recerca.clinic.cat (V.P.-R.); laragons@recerca.clinic.cat (L.A.-S.); flozano@clinic.cat (F.L.); 2 Department de Biomedicina, Facultat de Medicina, Universitat de Barcelona, 08036 Barcelona, Spain; jade.liu.tindall@gmail.com; 3 Sepsia Therapeutics S.L., 08902 Barcelona, Spain; misamat@sepsia.com; 4 Catalan Institution for Research and Advanced Studies (ICREA), 08010 Barcelona, Spain; toni.gabaldon@bsc.es; 5 Barcelona Supercomputing Centre (BSC-CNS), 08034 Barcelona, Spain; 6 Institute for Research in Biomedicine (IRB Barcelona), The Barcelona Institute of Science and Technology, 08028 Barcelona, Spain; 7 CIBER de Enfermedades Infecciosas, Instituto de Salud Carlos III, 28029 Madrid, Spain; 8 Servei d’Immunologia, Centre de Diagnòstic Biomèdic, Hospital Clínic Barcelona, 08036 Barcelona, Spain

**Keywords:** mycobiome, immune cells, fungal receptors, cancer, infection, inflammation, autoimmunity

## Abstract

The fungal component of microbiota, known as the mycobiome, inhabits different body niches such as the skin and the gastrointestinal, respiratory, and genitourinary tracts. Much information has been gained on the bacterial component of the human microbiota, but the mycobiome has remained somewhat elusive due to its sparsity, variability, susceptibility to environmental factors (e.g., early life colonization, diet, or pharmacological treatments), and the specific in vitro culture challenges. Functionally, the mycobiome is known to play a role in modulating innate and adaptive immune responses by interacting with microorganisms and immune cells. The latter elicits anti-fungal responses via the recognition of specific fungal cell-wall components (e.g., β-1,3-glucan, mannan, and chitin) by immune system receptors. These receptors then regulate the activation and differentiation of many innate and adaptive immune cells including mucocutaneous cell barriers, macrophages, neutrophils, dendritic cells, natural killer cells, innate-like lymphoid cells, and T and B lymphocytes. Mycobiome disruptions have been correlated with various diseases affecting mostly the brain, lungs, liver and pancreas. This work reviews our current knowledge on the mycobiome, focusing on its composition, research challenges, conditioning factors, interactions with the bacteriome and the immune system, and the known mycobiome alterations associated with disease.

## 1. Introduction

The human body is a complex ecosystem hosting symbiotic, commensal, and pathogenic microbial relationships. The human microbiota, defined as the consortium of living microorganisms that reside in the human body, differs between individuals and body sites. It is subject to constant change, as its diversity is influenced by the environment of each body compartment. The microbiome refers to the collection of microbial genomes, metabolites, and structural elements within the microbiota [1]. So, each body compartment, such as the skin and the respiratory, gastrointestinal (GI), and genitourinary tracts, presents its own specific microbiome profile [2]. Most studies focus on the bacterial communities that constitute the majority of the human microbiota, but other lesser-studied microorganisms, such as fungi, archaea, viruses, and protists, also participate in that ecosystem with an impact on health and physiological homeostasis. In this context, the mycobiome, namely the fungal component of the microbiome, has recently gained much interest for its implications in metabolic disorders, autoimmunity, and cancer.

This review explores the mycobiome-immune axis with a particular focus on the immunomodulatory properties of fungal communities, the underlying mechanisms, and the impact of this interaction in health, disease, and in fungal-based therapeutic strategies.

## 2. Components of a Healthy Mycobiome

The current lack of consensus on what constitutes a healthy mycobiome is due to its sparsity compared to the bacteriome, its susceptibility to external factors, and its temporal, interpersonal, and intrapersonal variability [3,4]. This, together with specific methodological challenges, results in under-exploration and limited data at hand on the human mycobiome. This section will deal with the mycobiome details we have so far for the different human fungal niches. A summary of the most frequent taxa in each body niche is displayed in Table 1.

### 2.1. Gastrointestinal Tract

The GI microbiota has been extensively studied. Gut microbiota colonization varies throughout the GI tract in a continuum of increasing microbial densities, with the stomach and the duodenum being less populated segments, gradually increasing in the jejunum and ileum, and culminating with the highest concentration in the colon [5].

The Human Microbiome Project (HMP) [6] investigated mycobiome composition in a large cohort of healthy individuals through fungal Internal Transcribed Spacer 2 (ITS2) and 18S rRNA sequencing, revealing fungi mainly from the Ascomycota and Basidiomycota phyla in fecal samples [4]. It was suggested that both phyla, which are also dominant in skin and vaginal microbiota, posed an advantage in mammalian hosts. Additionally, the project showed that the most prevalent fungal genera included *Saccharomyces*, *Malassezia*, *Candida*, *Cyberlindnera*, *Penicillium*, *Cladosporium*, *Aspergillus*, *Debaryomyces*, *Pichia*, and *Clavispora,* with a remarkable overall dominance of the *Saccharomyces* genus. Other studies, including smaller or disease-specific cohorts, also identified *Cryptococcus*, *Trichosporon*, and *Galactomyces* as common mycobiome participants in the gut [7]. Most studies are carried out on readily available fecal samples, but samples from the intestinal mucosa exhibit a more stable fungal community. The species *Rhodotorula mucilaginosa*, *Galactomyces geotrichum*, *Candida albicans*, *Candida dubliensi*, *Septoria epambrosiae*, *Cladosporium carnescens*, *Cladosporium cladosporioides*, *Bulleracrocea* spp., *Raciborskiomyces longisetosum*, and *Penicillium ialicum* are also detectable in this setting [8].

### 2.2. Oral Cavity

The oral cavity mycobiome, one of the most versatile microbiomes, holds significant importance since disruption of the oral microbiome often underlies oral disease, especially in immunocompromised individuals [9]. The predominant fungal phyla in the oral cavity include Ascomycota, Basidiomycota, Glomeromycota, and Mucoromycota [10], with a high prevalence of the genus *Candida* from the phylum Ascomycota showing, furthermore, an age-dependent abundance. Most studies also include *Cladosporium*, *Aureobasidium*, and Saccharomycetales as frequently identified taxa with *Aspergillus*, *Fusarium*, and *Cryptococcus* present in lower frequencies [11].

### 2.3. Respiratory Tract

In the respiratory tract mycobiome, the conducting segment (from nose to bronchi) is constitutively colonized, whilst the respiratory portion (from bronchioles to alveoli) remains predominantly sterile or with minimal microbial load [2]. The lung mycobiome from healthy individuals predominantly comprises *Aspergillus* species, and this presence is strongly associated with environmental factors [12]. Other predominant genera include *Cladosporium*, *Eurotium*, and *Penicillium* [13,14], but in other cohorts, *Candida* and *Saccharomyces* are also highlighted as relevant groups [15].

### 2.4. Skin

The skin is colonized by numerous commensal microorganisms, of which 10% correspond to fungal species [16]. The composition of the skin mycobiome varies depending on gender, age (changing greatly during puberty), and body site (due to relative humidity variations) [2,10]. As in the GI tract, Ascomycota and Basidiomycota are the main resident phyla, with the basidiomycetous yeast *Malassezia* being the most recurrent skin colonizer, located at the inner side of the elbows, back, outer auditory canal, nostril, and some facial areas [17,18].

### 2.5. Genitourinary Tract

The urinary tract of healthy individuals is predominantly sterile except for the urethra, which is significantly more colonized in women [10]. Preliminary characterization of urinary fungal communities from healthy volunteers showed vast fungal interindividual variability, rendering the identification of a single dominant taxon inviable [19]. Further research is henceforth required to establish a specific profile of the urinary mycobiome beyond the more commonly known urinary tract infections (UTIs).

Conversely, the vaginal microbiome is largely assembled by bacterial and fungal species, mostly *Lactobacillus* and *C. albicans*, respectively. Whereas *C. albicans* commonly colonizes the vaginal lumen asymptomatically, symptomatic infection can result from overgrowth, leading to vulvovaginal candidiasis (VVC), the most prevalent human *Candida* infection [20]. Other taxa such as Saccharomycetales, Davidiellaceae, *Cladosporium*, and *Pichia* are also present in the vaginal mycobiome [21,22].

**Table 1:** Most frequent taxa present on each body compartment.

Body Niche	Frequently Reported Fungal Taxa	Relative Abundance	Study
Gastrointestinal tract	*Saccharomyces cerevisiae*	13.9%	[23]
	*Candida* spp.	0.5%	
	*Penicillium*	<1%	
	*Aspergillus*	25.7%	
	*Debaryomyces udenii*	19.9%	
	*Cladosporium*	6.1%	[24]
	*Pichia*	18.9%	
Oral cavity	*Malassezia* spp.	37.7%	[25]
	*Epicoccum* spp.	33.8%	
	*Candida/Pichia* spp.	9.6%	
	*Cladosporium/Davidiella* spp.	3.0%	
	*Alternaria* spp.	1.9%	
Respiratory tract	Saccharomyctes	48.1%	[12]
	Microsporidia	23.6%	
	Dothideomycetes	16.6%	
	Cryptomycota	3.0%	
	Chytridiomyctes	3.0%	
Skin	*Malassezia restricta*	89.5%	[26]
	*Malassezia globosa*	2.90%	
Vaginal tract	*Candida albicans*	63.50%	[27]
	*Leptosphaerulina chartarum*	9.90%	
	*Clavispora lusitaniae*	9.10%	

## 3. Methods and Limitations in Mycobiome Research

The significance of the human mycobiome cannot be understated, but admittedly, it has received limited research attention compared to bacterial communities. This is illustrated by the fact that less than 3% of the total microbiome research deals specifically with its mycobiome component [28], which is, in turn, a direct consequence of the limitations inherent to the taxonomic methods routinely applied.

Traditionally, fungal composition has been studied with culture-dependent methods such as biochemical assays, microscopy, and growth observations in cultures [29]. This allowed comprehensive phenotypic characterization and the isolation of strains of interest, but it was severely limited by the so-called “culturability bias”, resulting in a significant underestimation of true fungal diversity and an overrepresentation of fast-growing, culturable species that were viable in the chosen growth conditions (media, temperature, oxygen) [30]. This approach was also labor-intensive and time-consuming, limiting the number of samples that could be analyzed. The advent of molecular techniques, particularly DNA sequencing, revolutionized mycobiome studies by enabling culture-independent assessment of fungal diversity. PCR-based amplicon sequencing of the Internal Transcribed Spacer (ITS) region of the ribosomal RNA (rRNA) gene cluster is the most widely adopted approach [31], together with 18S rRNA sequencing. The ITS approach detects both culturable and unculturable fungi, providing a more comprehensive view of the fungal diversity in a sample. Next-generation sequencing (NGS) and bioinformatics analysis have enabled large-scale studies and the simultaneous analysis of thousands of samples. However, there are no truly universal primers for ITS. Existing primer pairs are designed to amplify a broad range of fungi, but they still exhibit biases, preferentially amplifying certain taxa over others, leading to an inaccurate representation of community composition. A particular problem is that the ITS region varies in length and copy number across fungal species, complicating accurate quantification and taxonomic assignment. The resolution provided by ITS sequencing is adequate at the genus level, but the accuracy is inherently dependent on the quality and completeness of reference databases (e.g., UNITE, NCBI GenBank). Additionally, the identification of cryptic species, defined as species that are indistinguishable in terms of morphology but genetically different [32], represents a major challenge for ITS. These sibling or cryptic species usually differ in only a few nucleotide positions, and these small differences can be wrongly attributed to intragenomic variation, leading to misidentification [33]. Thanks to recent drops in sequencing costs and advanced bioinformatics tools, a larger number of studies now use whole-genome shotgun (WGS) sequencing that interrogate a complete DNA set in a sample. This approach provides a more unbiased quantification and offers the potential to detect the presence of all microorganisms in a sample, including fungi, alongside bacteria, archaea, protists, and viruses. Furthermore, it offers better taxonomic resolution and enables the inference of functional capabilities. However, the low fungal content in the human microbiome continues to be a hurdle, requiring deeper sequencing to obtain sufficient fungal reads for robust analysis, making WGS sequencing less accessible for large-scale mycobiome profiling compared to amplicon sequencing. Analyzing large WGS datasets is computationally complex and labor-intensive, requiring substantial computing power and specialized bioinformatics pipelines [34]. A particularly challenging limitation in mycobiome WGS is the incomplete fungal species reference databases, far less comprehensive than bacterial ones [35]. This hinders accurate taxonomic assignment of novel or less-studied fungi and limits the functional annotation of their genes.

Accurate characterization of the mycobiome also hinges critically on our ability to extract and quantify fungal DNA. The presence of a thick fungal cell wall composed of chitin, glucans, and mannoproteins constitutes a barrier for DNA extraction [36], often requiring specific DNA extraction protocols to ensure efficient and unbiased fungal DNA. Inadequate cell lysis can result in an overall underrepresentation of fungi with respect to other microbes, or to certain fungal taxa. For instance, DNA extraction protocols may vary in yield efficiency on yeast or filamentous fungi, leading to a skewed perception of community composition and diversity. Another important challenge is the typically scarce fungal proportion of the total microbial biomass in the human microbiome. This means that fungal DNA is often present in minute quantities relative to bacterial or host DNA, which means fungal DNA is subject to higher stochastic variations and is harder to quantify precisely. To mitigate these challenges, a combination of mechanical, enzymatic, and chemical lysis methods is typically employed. Mechanical lysis, such as bead-beating with appropriately sized and dense beads (e.g., zirconia/silica beads), is often crucial for physically disrupting tough fungal cell walls. Optimization of bead-beating parameters (speed, duration, number of cycles) and enzyme concentrations is critical for maximizing yield and minimizing bias. The use of commercial kits can streamline the process, but even these often require user-specific adaptations and rigorous quality control, including the use of positive (mock communities) and negative controls, to ensure accurate representation of the fungal community [37].

The mycobiome composition is likewise affected by factors such as age, diet, host immunity, genetics, and interactions with other microorganisms, leading to diversity and stability variations [29]. In this sense, discriminating between foodborne fungi contaminants and commensal fungi that colonize the gut would benefit from specific tools and biomarkers (e.g., procalcitonin and neutrophil count) [29] beyond a clinical criterion that recognizes colonizers as asymptomatic.

## 4. Factors Influencing Mycobiome Composition

Many factors continuously shape mycobiome composition, including diet, antibiotics, antifungals, and other drugs, as well as host factors such as individual genetics, age, or immune status. Subsequently, fungal analyses also need to be studied in a sociodemographic population context [38].

Gut mycobiota colonization starts at birth with an increase in fungal diversity during childhood, which decreases at the start of adulthood [28] (Table 2). Neonatal gut mycobiome colonization is mainly determined by the delivery mode. Vaginally delivered infants acquire vaginal fungal colonizers (e.g., *Candida* and *Pleosporales*) and a more diverse mycobiome, whilst those delivered by caesarean section acquire skin-related fungi (e.g., *Malassezia*) [39,40]. The feeding method also modulates colonization, as detectable levels of *Malassezia*, *Davidiella*, *Sistotrema*, and *Penicillium* have been commonly found in breast milk [38]. The influence of breastfeeding on mycobiota composition has recently become an active area of research.

After colonization and in the span of a lifetime, different factors shape the mycobiome, with an important role attributed to dietary habits [44]. In this context, high levels of *Candida* correlate with carbohydrate intake, whereas *Aspergillus* spp. becomes scarce following short-chain acid consumption [45]. It has also been reported that plant-based diets lead to increased *Candida* spp., whereas animal-based ones correlate with enriched *Debaryomces* spp. and *Penicillum* spp. [46].

In terms of sex differences, the increased abundance and diversity of fungal isolates in females, as assessed by culture-based techniques [47], are corroborated by separate mycobiome clusters from female and male samples following metagenomic analysis, suggesting a sex bias in fungal communities that may reflect hormonal or diet-based influences.

In line with general microbiota, other factors in addition to hormones or diet, such as body mass index (BMI), occupation, autoimmune diseases, and the use of probiotics, are known to impact mycobiome composition [44].

## 5. The Mycobiome’s Interactions

The microbiota can be seen as an intricate system where all its components, including the mycobiota, engage in continuous interactions among themselves and with the host to maintain homeostasis. A disruption to this delicate balance may lie behind a number of diseases, as discussed in Section 6. But let us first look at the nature of the mycobiome interactions with bacteria and the immune system.

### 5.1. Interactions Between Fungi and Bacteria

Studying the interactions between fungi and bacteria generally involves the induction of gut dysbiosis by antifungal or antibacterial treatments. The mouse model of colitis induced by dextran-sulfate-sodium serves as a good example of the impact of these relationships, where antifungal drugs reduce fungal diversity while promoting pathogenic bacteria and exacerbating inflammation [48]. Contrary to this, when an antibiotic is used to induce dysbiosis, commensal fungi, including *C. albicans* and *S. cerevisiae*, functionally replace the bacteriome [49].

The bacteriome–mycobiome relationship can also provide health benefits. This is illustrated by the secretion of enzymes from *Saccharomyces boulardii* that inhibit *Clostridium difficile* [50] and *Escherichia coli* toxins [51]. Similarly, fatty acid metabolites secreted by commensal bacteriome impair the TOR pathway that normally activates *C. albicans* hyphae formation and colon invasion [52]. It is also widely acknowledged that lactic acid production by *Lactobacillus* species can protect from vaginal candidiasis [53,54,55].

Interactions between fungi and bacteria can also impact metabolic disorders, which occasionally exert negative effects on the host, as would be the case for the survival of *Helicobacter pylori* in the stomach’s acidic pH by residing within vacuoles in *C. albicans* cells [56].

The case of mixed fungal–bacterial biofilms (MFBBs) illustrates a different type of interaction that occurs in infectious keratitis between *Staphylococcus aureus* and *Aspergillus* fumigatus [57], when the fungus serves as a carrier to *S. aureus*, facilitating epithelial penetration [58]. Other MFBBs occur in the oral cavity between *C. albicans* and *Streptococcus*, typically associated with dental caries and more severe oropharyngeal pathologies [59], where SspA and SspB streptococcal adhesins interact with the Als3 protein from C. *albicans* to promote colonization [60]. Mutualistic relationships in MFBBs examples are abundant, including in vitro formation of biofilms between *Trichosporon asahii* and *Staphylococcus simulans*, *C. albicans*, and *Citrobacter freundii* [61] or *Candida tropicalis*, *Serratia marcescens*, and *E. coli* [62], where bacterial lipopolysaccharide production enhances fungal biofilm maturation [63,64].

### 5.2. Mycobiome Interactions with the Immune System

**Figure 1:**
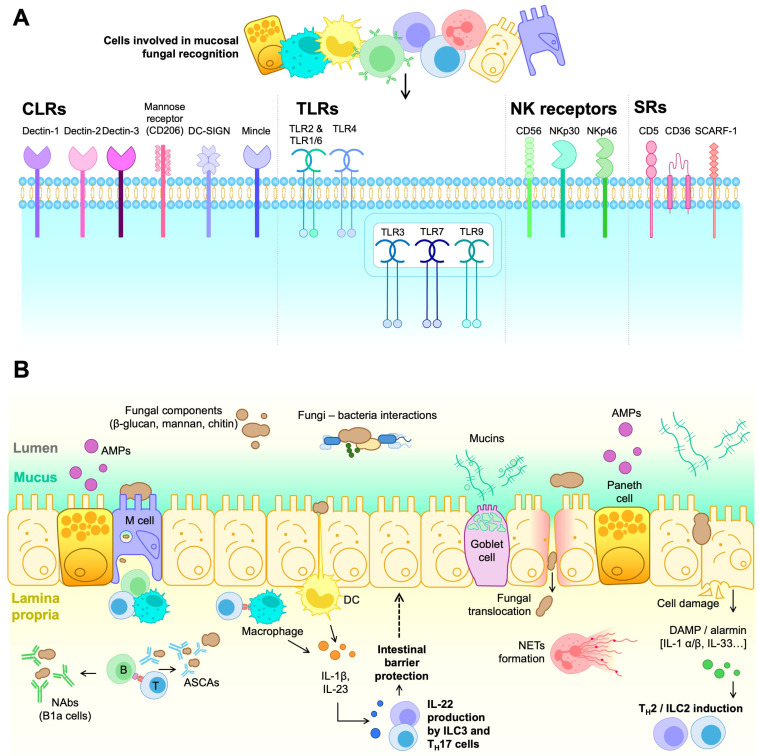
Immune cell receptors and cell-mediated responses contributing to fungal recognition and overgrowth control in the intestinal mucosa. (**A**) Structurally and functionally diverse families of pattern recognition receptors (PRRs) expressed by different innate and adoptive immune cell types well-represented in the intestinal mucosa and involved in fungal recognition (i.e., CLRs, TLRs, NKRs, and SRs). (**B**) Cell-mediated responses against fungi first involve different cell types from the intestinal mucosal barrier, including enterocytes, Goblet cells, Paneth cells, and M cells involved in alarmin release, mucin production, AMP production, and transcytosis processes (sample and transport antigens/pathogens from the luminal surface to the sub-epithelium), respectively. In the lamina propria, DCs and macrophages can engage in fungal antigen presentation to T cells and participate in the antifungal response via cytokine secretion and phagocytosis of harmful fungal particles. In this context, T_H_17 and ILC3 responses can be triggered, ultimately leading to IL-22 release that can contribute to the integrity of the intestinal barrier. On the other hand, alarmins (e.g., IL-1β, IL-33) and DAMPs released in response to epithelial cell aggression or damage can trigger T_H_2 and ILC2 responses. Additionally, exposure to whole fungal cells and or particles can engage antifungal responses involving neutrophil activation (NETs formation) as well as T cell-dependent and independent B cell activation (production of ASCAs and NAbs by conventional or innate-type B1a cells, respectively).

A significant part of the mycobiome’s impact relies on specific interactions with the immune system, leading to beneficial or deleterious effects in the host, as schematically depicted in Figure 1.

In general, fungal recognition by the immune system relies on germ-line encoded receptors, the so-called pattern recognition receptors (PRRs). PRRs bind to conserved structures shared by different microbes and absent from the host. The PRR-ligand complex triggers immediate inflammatory responses (danger signals), contributing to the subsequent activation of innate and adaptive immune responses. The best-characterized PRRs in antifungal immunity are the C-type lectin receptors (CLRs), primarily expressed in monocytes, macrophages, dendritic cells (DCs), and granulocytes [65]. In this context, Dectin-1 stands out, as it specifically recognizes the β-1,3-glucans in fungal cell walls [66]. Particularly, Dectin-1 co-localizes and interacts with the Toll-like receptor 2 (TLR2), another membrane-bound PRR, synergistically over-activating both receptors to turn on tyrosine phosphorylation [67,68]. Additionally, Dectin-1 associates with Galectin-3 (a soluble PRR) in macrophages, which mediates discrimination between pathogenic strains such as *C. albicans* and non-pathogenic ones such as *S. cerevisiae* [69].

Mannose polymers are recognized by CLRs such as Dectin-2, Mannose receptor (MR), Mannose-binding lectin (MBL), Mincle, and DC-SIGN (for Dendritic Cell-Specific Intercellular adhesion molecule-3-Grabbing Non-integrin) [70]. In this context, Dectin-2 also heterodimerizes with Dectin-3 to bind α-mannans more effectively during fungal infections [71].

Downstream of several C-type lectin receptors (CLRs), we can find caspase recruitment domain-containing protein 9 (CARD9), a cytosolic adaptor molecule critical in antifungal immunity. Upon receptor engagement, CARD9 promotes the activation of the nuclear factor-κB (NF-κB) and mitogen-activated protein kinase (MAPK) pathways, which in turn promote the production of pro-inflammatory cytokines and reactive oxygen species (ROS), as well as the formation of neutrophil extracellular traps (NETs) [72]. NETs contain neutrophil nuclear DNA and histones that trap and kill extracellular pathogens by phagocytosis. Additionally, CLR’s signaling regulates T cell activation and differentiation through various processes: (i) antigen recognition and presentation to prime naïve CD4^+^ T cells via class II major histocompatibility complex (MHC-II) molecules; (ii) induction of co-stimulatory molecules (e.g., CD40, CD80 and CD86) that support peptide-MHC/TCR-mediated activation; and (iii) release of cytokines and chemokines that promote CD4^+^ T cell differentiation into specific effector helper (T_H_) and regulatory (Treg) T cell subsets [73].

Toll-like receptors (TLRs) constitute another structurally different group of PRRs, some of which recognize fungal structures and trigger intracellular signals through adaptor proteins such as myeloid differentiation primary response 88 (MyD88) and TIR-domain-containing adapter-inducing IFN-β (TRIF) [74]. TLRs are broadly distributed across tissues and can be found in innate immune cells (e.g., monocytes, macrophages, neutrophiles, DCs, natural killer cells (NK), mast cells, eosinophils), non-hematopoietic cells (epithelial and endothelial cells, adipocytes, fibroblasts), and adaptive immune cells (T and B lymphocytes) [75]. TLR’s anti-fungal immune responses depend on the contact with fungal components and indirect association with other PRRs. For example, TLR3, TLR7, and TLR9 sense fungal RNA and DNA, leading to neutrophil recruitment, cytokine production, and clearance of pathogenic fungi [76], whilst TLR2 and TLR4 sense zymosan (a β-glucan- and mannan-rich particle derived from *S. cerevisiae*) and glucuronoxylomannan (GXM) from *C. neoformans*’s capsule, respectively, and couple with the MyD88 pathway and activate NF-kB and mitogen-activated protein kinase (MAPK), which induce IL-12, TNF-α, TGF-β, and IFN-γ production [77].

Beyond the roles of CLRs and TLRs in fungal recognition, other immune receptors contribute to antifungal defense. NK cells’ CD56, NKp30, and NKp46 receptors sense fungal components to initiate immune responses. The CD56 molecule interacts with *A. fumigatus* through galactosaminogalactan recognition [78], while the NKp30 and NKp46 members of the natural cytotoxicity receptor (NCR) family recognize β-1,3-glucans and fungal adhesins, respectively [79,80]. This interaction activates NK cells, cytotoxicity, and cytokine production to eliminate fungal pathogens, complementing the function of CLRs and TLRs in maintaining host defense.

Scavenger receptors (SRs), another group of PRRs, recognize fungi alongside TLRs, triggering phagocytosis, intracellular signaling, and inflammatory cytokine and chemokine production. Specifically, the CD5 SR on all T cells and some B cells, macrophages, and DC subsets, interacts with β-1,3-glucans in the fungal wall with a similar affinity to Dectin-1 [81]. The immunotherapeutic use of soluble human CD5 (shCD5) in mouse models of *C. albicans* and *Cryptococcus neoformans* fungal sepsis increased survival, concomitantly with reduced fungal loads, increased IFN-γ expression, and leukocyte infiltration in target organs (namely, spleen and kidney for *C. albicans*, lung and brain for *C. neoformans*) [82]. Moreover, the addition of shCD5 to ex vivo *C. albicans*-splenocyte co-cultures increased IFN-γ and TNF-α production, activating macrophage and neutrophil antifungal cytotoxic responses [82].

CD36 and SCARF1 (for Scavenger receptor class F member 1; also named SREC-1 or SR-F1) are type I transmembrane SRs that recognize multiple endogenous and exogenous ligands such as modified low-density lipoproteins (LDLs). They are mainly expressed in macrophages, DCs, and endothelial cells and act as co-receptors for TLR2 signaling in antifungal responses [83]. Notably, both molecules are essential for *C. neoformans* and *C. albicans* infection to mediate cytokine response and macrophage binding [84].

Fungal components first encounter PRRs at the mucocutaneous epithelial barrier and associated lymphoid system. In the skin, the epidermal layer, made up of dead keratinocytes and inert hydrophobic components, maintains skin homeostasis [85]. This layer is underlined by the *stratum granulosum*, characterized by tight junctions between keratinocytes renewed by the stem cells hosted at the *stratum basale*. The dermal extracellular matrix harbors nerve fibers and vascularization to enable immune cell infiltration during fungal infection. In addition to acting as a physical barrier, epithelial cells engage in antifungal responses by producing both anti-microbial peptides (AMPs) with fungitoxic capacity and pro-inflammatory cytokines [86], including IL-1α, IL-1β, IL-8, G-CSF, and GM-CSF in responses against *C. albicans.* Additionally, Langerhans cells present in the epithelial layer function as antigen-presenting cells (APCs) to generate antigen-specific T_H_17 cells upon *C. albicans* infection [87]. Dermis-resident immune cells include macrophages, mast cells, and γδ and αβ T cells. Specifically, γδ T cells have a precise role as the source of IL-17A in response to infection by the commensal *Malassezia* yeast [88].

In the case of mucosae from GI, urogenital, and respiratory tracts, AMPs and mucins secreted by goblet cells act as the first line of defense at the epithelial barrier [89]. Additionally, Microfold (M) cells—a highly specialized epithelium in luminal antigen phagocytosis and transcytosis—act as the entry portal for *C. albicans* into the intestinal epithelial barrier by specifically invading these cells [90]. Despite not showing specific degranulation upon fungal stimuli [91], Paneth cells can engage in intestinal fungal recognition by releasing specific AMPs, such as the YY peptide for *Candida* spp. inhibition [92,93].

However, the key drivers of fungal immunity in the mucosae are mononuclear phagocytes (MNPs), especially those expressing CX3CR1 involved in T_H_17 and IgA responses, and infiltrating neutrophils, which engage in phagocytosis and neutrophil extracellular trap (NET) formation [94]. Fungal antigen recognition by macrophages via Dectin-3 maintains homeostasis with the intestinal commensal mycobiota. Specifically, mice lacking Dectin-3 present an increased fungal burden that induces glycolysis in macrophages, subsequently leading to IL-7 secretion. IL-7 overproduction enhances IL-22 secretion by group 3 Innate Lymphoid Cells (ILC3s), which ultimately promote colon cancer, highlighting eubiosis and optimal fungal recognition in disease [95]. Conversely, IL-22 may benefit fungal response and homeostasis. Specifically, mucosa-associated fungi (MAF) reduce intestinal permeability and protect against intestinal injury thanks to IL-22 secretion by T_H_17 and ILC3 cells [96]. The role of ILCs in mucosal immunity has been described [97]. While ILCs include several subtypes (i.e., ILC1, ILC2, and ILC3), only ILC3 is involved in fungal responses. Thus, IL-23 and IL-1β secretion by CD11c^+^ myeloid DCs leads to the production of IL-22 by ILC3, which helps maintain intestinal barrier integrity [98].

In any case, failed PRR responses lead to cell destruction, with damage-associated molecular patterns (DAMPs) and alarmins signaling other responses and amplifying inflammation. These alarmins include protein and non-protein structures such as IL-1β and IL-1α, essential in various invasive fungal infections [99,100]. Additionally, the alarmin IL-33 is known to stimulate IL-4, IL-5, and IL-13 production by ILC2 cells, contributing to fungal-derived allergic inflammation [101].

Fungi like *S. cerevisiae* and *C. albicans* notably regulate the immune response by inducing trained immunity (innate immune memory) and reprogramming monocytes, all of which maintain gut homeostasis and protect against invading pathogens [29]. Specifically, *C. albicans* is recognized by macrophages via CLRs and stimulates T_H_17 responses, inducing T_H_17 cells and circulating IL-17-reactive neutrophils [76]. This response helps memory T cell formation that cross-reacts with species that share common T cell receptor (TCR) sequences, such as *Candida* and *S. aureus*, consequently blocking the invasion by exogenous forms of these microorganisms. In addition, T_H_17 cells induced by *C. albicans* in the gut can be expanded in the lungs through cross-reactivity to *A. fumigatus* [73].

Similarly, the mycobiome can modulate B cell activity to recognize fungal particles in a T cell-dependent manner (via BCR and CD40/CD40L co-stimulation) or in a T cell-independent manner via TLR recognition [102]. *S. cerevisiae* and/or *C. albicans* induce anti-*S. cerevisiae* IgA and IgG antibody production (ASCAs), that mediate fungal pathogen clearance via mannan recognition [103]. Additionally, B cells promote MHC-II-restricted antifungal T_H_17 responses in an antibody-independent manner [104]. Interestingly, CD5-expressing B1 cells produce polyreactive natural antibodies (NAbs) against fungal molecules and engage in macrophage-induced opsonization [105].

## 6. The Gut Mycobiome in Health and Disease

The gut mycobiome determines protection or susceptibility to disease by helping configure the host’s immune system and therefore, dampening or promoting inflammatory responses in a niche-dependent mode.

### 6.1. GI Tract Diseases

Inflammatory bowel diseases (IBDs), including UC and Crohn’s disease (CD), have been associated with gut mycobiota dysbiosis. As previously stated, *C. albicans* modulates T_H_17 responses, which are normally limited to the intestines and the skin under homeostatic conditions, thus contributing to local inflammatory disorders (e.g., IBD) when dysregulated [106].

In fecal samples from IBD patients, the *C. albicans* abundance doubled in relation to controls, increasing concomitantly with disease flares and reversed during remissions, suggesting an inter-dependent co-relationship with the microorganism [107]. Other findings highlight the connection between IBD and mycobiome dysbiosis. High levels of antibodies against fungal cell wall sugars and reduced IgG responses were observed in CD carriers of a mutated form of the chemokine receptor CX3CR1, and *Debariomyces hansenii* expansion in the mucosal wounds of these patients impaired their healing [108]. Moreover, single-nucleotide polymorphisms (SNPs) of genes involved in fungal recognition in humans (e.g., CLEC7A encoding Dectin-1 and CARD9, both involved in T_H_1 and T_H_17 cell differentiation) [109], and genetic ablation of CX3CR1 in mouse mononuclear phagocytes (needed for initiating antifungal immune responses) were correlated with worse UC [110]. Furthermore, the fungal/bacterial diversity ratio increases in samples from CD patients, suggesting that the GI environment of these patients is better suited to fungal colonization [111,112]. CARD9 protects against pathogenic fungi and also regulates the normal balance of the mycobiome. This adaptor molecule has been involved in the control of gut microbiota metabolism, and its deficiency leads to shifts in microbiota composition and impaired immune signaling, affecting susceptibility to inflammatory bowel diseases (IBD) [113].

The presence of mycotoxins, secondary metabolites produced by fungal species such as *Aspergillus*, *Penicillium*, and *Fusarium*, has also been related to alterations in gut microbiota composition, often leading to dysbiosis and promoting IBD pathogenesis [114].

It is worth noting that *S. cerevisiae* relieves gastroenteritis and adherent-invasive *E. coli*-induced colitis in mice and also alleviates IBD abdominal pain in humans [115,116].

Celiac disease is also associated with mycobiome dysbiosis. *C. albicans* hyphal wall protein resembles the T cell gliadin epitopes and is a transglutaminase substrate [117], so its presence increases the production of anti-gliadin and anti-transglutaminase antibodies, becoming a potential causal trigger for the disease under chronic inflammation conditions. However, when homeostasis is reconstituted, *C. albicans* interaction with mast cells can alleviate celiac symptomatology [118].

### 6.2. Neurological Disorders

Multiple neurological disorders have been associated with the mycobiome, with research suggesting a potential link between mycobiome dysbiosis and schizophrenia, autism, and multiple sclerosis.

Gut microbiota influences brain functions through the autonomic nervous system, cytokines, metabolic products (e.g., tryptophan, GABA, and acetylcholine), and by modulating the hypothalamus-pituitary-adrenal axis. This gut–brain connection has been correlated to depression, autism, Parkinson’s disease, Alzheimer’s disease, and eating disorders [119].

Some findings, like *Candida’*s ability to regulate this axis [120], lead to the hypothesis that gut fungi could favor mental disease progression. In this regard, *Candida kefyr* alleviated experimentally induced autoimmune encephalomyelitis in mice [121], schizophrenia patients presented changes in gut mycobiome composition and diversity [122,123], and neurons containing fungal cells have been observed in patients with Alzheimer’s disease [124].

### 6.3. Lung Disorders

Gut mycobiome dysbiosis has been widely associated with allergic airway disease and pulmonary pathologies [125,126]. The link between the gut mycobiome and the lungs relates to cell migrations between the two organs of either fungi-primed immune cells (via Th2 and T_H_17 responses) or gut fungi. Additionally, antibiotic-induced dysbiosis can lead to *Candida* overgrowth in the gut in an opportunistic manner, inducing T_H_17 responses and macrophage differentiation in the airways, all of which increase lung and airway inflammation [103,108].

Gut mycobiome disequilibrium has been correlated with Cystic Fibrosis (CF) and infection by Influenza A virus, in which *C. albicans* and/or *S. cerevisiae* intestinal colonization protected against respiratory epithelium cell invasion [127].

There is growing concern about mucormycosis, a severe infection caused by members of the Mucorales order [128]. Emerging infections by these fungi, mainly in immunocompromised individuals, show high mortality rates (>40%), with higher incidence following the SARS-CoV-2 pandemic [129]. Mucormycosis is mainly caused by mucorales species, of which *Rhizopus* species account for >70% of all cases, but *Mucor* and *Lichtheimia* species are also frequent etiologic agents [130]. There is much preoccupation with mucormycosis in patients on antifungal treatment as a result of hypervirulent strains following epigenetic modifications [131,132].

### 6.4. Systemic Infections

Patients experiencing chronic critical illness (CCI) show an altered gut mycobiome profile up to two weeks after experiencing sepsis [133]. Relevant relationships between gut mycobiome composition and viral infections have been described. For instance, *Geotrichum* species are more represented in SARS-CoV-2 patients than in controls [134], *C. albicans* or *S. cerevisiae* can reverse susceptibility of Influenza A infection upon antibiotic treatment in mice [49], and in the case of Human immunodeficiency virus type 1 (HIV-1)/Hepatitis C virus (HCV) co-infected patients show decreased fungal alpha diversity, and co-infection was specifically associated with a more dysbiotic mycobiome composition [135].

Invasive pulmonary aspergillosis, caused by *Aspergillus*, has been recognized as an important opportunistic infection in patients with viral pneumonia [136]. This condition, which affects critically ill patients after ICU admission, has been related to two specific viral conditions: influenza-associated pulmonary aspergillosis (IAPA) and COVID-19-associated pulmonary aspergillosis (CAPA). IAPA presents a variable incidence (10 to 30%), and its appearance on patients occurs within a short time after ICU admission (median of 3 days). However, despite the CAPA incidence being similar (4–35%), its appearance mostly occurs 7 days after the ICU admission [137,138]. Both present high rates of mortality (40–60% and 60–70%, respectively), and both share airway epithelial damage caused by the viral infection [136]. Little is known about the alterations that these conditions produce in gut mycobiota, but COVID-19 and H1N1 influenza patients present lower gut fungal α-diversity and microbial diversity [139,140].

Finally, *Candida auris* is a multidrug-resistant infectious fungus that represents a current threat to public health. Its virulence relies on its cell polymorphism, environmental adaptation, and endurance on surfaces, as well as on its immune evasion mechanisms [141]. Specifically, innate immunity modulation has been associated with *C. auris* invasive candidiasis, showing enhanced resistance towards neutrophil activation and NET formation, macrophage evasion, and lower pro-inflammatory mediators production (IL-1β, IL-6, tumor necrosis factor-α, CXCL1/KC, and CXCL2/MIP2) [142].

### 6.5. Liver Disorders

Mycobiome dysbiosis has been linked to alcohol-associated liver disease [143] (F. Li et al., 2019). These patients show poor fungal diversity and richness, a correlation with *Candida* overgrowth, high expression of candidalysin (a hepatocyte-damaging *C. albicans* exotoxin), and high levels of serum ASCAs, suggesting increased systemic exposure to intestinal fungi or their products [144].

It is uncertain whether gut mycobiome alterations are a cause or a consequence of obesity, but some studies report diminished fungal diversity in obese patients, with *S. cerevisiae* and *C. albicans* reduced in obese children [145,146] and *Mucor* being more prevalent in non-obese subjects [144]. In this manner, mycobiome composition may be involved in the pathogenesis of non-alcoholic fatty liver disease, currently associated with obesity.

In light of the above, primary sclerosing cholangitis, another chronic liver disease, may be linked to the gut mycobiome, as it is frequently associated with IBD [147] and higher ASCA serum levels [148].

### 6.6. Pancreatic Disorders

The mycobiome may have a pathogenetic role in diabetes mellitus (DM), as type 1 and 2 DM (T1DM and T2DM) patients are largely colonized by *C. albicans*, and those with T1DM also present increased fungal diversity [149,150].

Furthermore, both bacteria and fungi are increased in the pancreas of patients with pancreatic ductal adenocarcinoma (e.g., *Malassezia*), perhaps as a consequence of their migration through Oddi’s sphincter [151]. The mycobiome may also signal fungal cell wall glycan (MBL and complement cascade), but it is unclear whether this represents the cause or consequence of malignant progression.

### 6.7. Skin Disorders

Skin mycobiome diversity tends to be low, but multiple studies found pathological relationships between these communities and dermatologic disorders [152]. In this context, *Malassezia* species stands out, being common in human skin as a commensal microorganism. In a study focused on atopic dermatitis (AD), these patients showed a different mycobiome composition with increased abundance of *Cladosporium*, *Malassezia*, *Candida*, *Diplodia*, *Saccharomyces*, *Penicillium,* and *Aspergillus* [153]. Interestingly, it was demonstrated that patients exposed to air-conditioning showed a mycobiome profile closer to that of AD rather than a healthy mycobiome.

On the other hand, the skin mycobiome profile of psoriasis patients receiving IL-23 inhibitors revealed increased species diversity after treatment [154]. Additionally, the abundance of *Malassezia* species decreased after the treatment. Outside this therapeutic context, the role of *Malassezia* in psoriatic patients has been widely discussed, but no definitive conclusion is in place. While some historic studies demonstrated patient improvement after antifungal treatment [155] or proved that *M. ovalis* fragments could induce scalp lesions mimicking psoriasis symptomatology [156], others demonstrated that there was no difference in scalp psoriasis development upon itraconazole treatment [157] or, more recently, that the relative abundance of *Malassezia* species is increased in healthy individuals compared to psoriasis patients [158]. On the contrary, in the case of pityriasis versicolor or tinea versicolor, it is well-known that *Malassezia* species are causative, with *M. furfur*, *M. globosa*, and *M. sympodialis* being the most common species [159].

### 6.8. Genitourinary Disorders

While urinary tract infections (UTIs) are usually associated with bacterial species, *Candida* infections represent 5–10% of diagnosed UTIs [160], and other genera, such as *Cryptococcus*, *Aspergillus*, *Histoplasma*, *Mucoraceae*, *Blastomyces*, and *Coccidioides*, can also be identified as the causative agents [161]. Candiduria, defined as the presence of *C. albicans* in urine, may occur in both asymptomatic and symptomatic UTIs [162].

Vulvovaginal candidiasis (VVC) caused by *C. albicans* is a prevalent disease of the lower female reproductive tract, where the microorganism exists in the mucosa asymptomatically [20]. While *C. albicans* is responsible for over 90% of VVC diagnoses, other non-albicans species can act as causal agents, such as *C. glabrata*, *C. krusei*, *C. parapsilosis,* or *C. tropicalis*. Interestingly, episodes of VVC are more common in the luteal phase of the menstrual cycle, when the levels of estrogen increase, inhibiting fungal opsonization and phagocytosis via complement impairment [163,164].

### 6.9. Cancer

The digestive system harbors the most diverse microbiome and mycobiome, so current fungal studies in tumor contexts are focused on this niche. The pancreas, considered both part of the digestive and the endocrine system, has gained interest thanks to the work of Aykut and colleagues [151]. These authors demonstrated that the infiltrating fungal composition of pancreatic ductal adenocarcinoma (PDA) was enriched for *Malassezia* species, and that oral antifungal treatment protected from tumor progression, suggesting a pivotal role of fungal species for both the nature and treatment of PDA. It was later demonstrated that mycobiome-driven tumorigenesis occurred via modulation of IL-33 secretion, which enhances T_H_2 and ILC2 recruitment and leads to enhanced tumor growth [165]. PDA has also been studied in association with the oral mycobiome, identifying *Aspergillus* and *Cladosporium* as putative biomarkers to distinguish PDAC patients when compared to healthy controls [166].

Gut mycobiome has also been extensively studied for colorectal cancer (CRC), showing an altered fungal profile for these patients, with increased presence of *Candida* and *Malassezia* and decreased presence of *S. cerevisiae* [167], together with a higher Basidiomycota:Ascomycota ratio [168]. Also, additional research highlighted the possible role of CARD9, a protein involved in Dectin-1 and Dectin-3 downstream signaling, in the mycobiome-CRC association. It was shown that *Card9^−/−^* mice displayed higher tumor burden and heavier fecal fungal load, and this was highly influenced by the housing conditions, suggesting a strong influence of horizontal mycobiome transfer [169].

In the case of gastric cancer, these patients show a specific disease signature, including decreased diversity and richness, higher proportion of opportunistic fungi, and increased Basidiomycota:Ascomycota ratio in concordance with CRC studies [170].

As for hepatocellular carcinoma, fungal aflatoxin (produced by *Aspergillus*) acts as a carcinogen by promoting mutations in the p53 tumor suppressor gene [171].

Mycobiome studies on salivary samples of oral squamous cell carcinoma (OSCC) patients in Sudan demonstrated that these individuals showed increased abundance of *Candida*, *Malassezia*, *Saccharomyces*, *Aspergillus*, and *Cyberlindnera* [172]. Specifically, whereas *Candida* was associated with poorer overall survival (OS), *Malassezia* pointed to favorable outcomes, to the extent of becoming an independent prognostic marker for OSCC. Another study confirmed the pivotal role of *C. albicans* in these patients and additionally identified other common non-albicans Candida (NAC) species in OSCC salivary samples, including *C. dubliniensis*, *C. tropicalis*, *C. glabrata*, *C. parapsilosis*, *C. sake*, *C. krusei*, and *C. guilliermondii* [173].

Enrichment of the *Blastomyces* genus was detected in lung cancer samples in a pan-cancer mycobiome analysis [174]. The analysis of the intratumor lung adenocarcinoma mycobiome detected a decrease in *Candida* and an increase in *Saccharomyces* in these patients [175]. Interestingly, it was later proven that a specific tumor-resident species, *Aspergillus sidowii*, mediated lung adenocarcinoma progression by modulating the activity of myeloid-derived suppressor cells (MDSC) [176]. It was shown that *A. sydowii* specifically promoted an immunosuppressing environment by stimulating IL-1β secretion via the β-glucan/Dectin-1/CARD-9 pathway, acting as a possible opportunistic causative agent.

### 6.10. Autoimmune Disorders

The relationship between mycobiome and pathological settings mostly relies on the mycobiome-immune axis. Thus, dysbiosis-derived imbalance of the immune system, or conversely, immune-derived dysbiosis, can be studied in the context of autoimmune diseases.

Central nervous system (CNS) samples from multiple sclerosis (MS) patients contained *Trichosporum mucoides,* otherwise absent from healthy controls, suggesting a link between CNS mycosis and MS-associated neuroinflammation [177]. Furthermore, when the gut mycobiome of MS patients was characterized, these subjects showed higher alpha diversity and an over-representation of *Saccharomyces* and *Aspergillus* [147]. Interestingly, while they found a positive association between *Aspergillus* and activated CD16^+^ DCs, *Saccharomyces* was positively associated with circulating basophiles and negatively with regulatory B (B_reg_) cells.

A study on fecal mycobiome profiles of systemic lupus erythematosus (SLE), undifferentiated connective tissue diseases (UCTDs), and rheumatoid arthritis (RA) patients demonstrated that the mycobiome composition varied widely between these patients and healthy controls [178]. Additionally, SLE patients displayed dysbiosis, specifically showing enrichment of the Pezizales, Cantharellales, and *Pseudaleuria* groups.

As for Sjögren syndrome, patients with a *Candida* infection had a higher risk of developing the disease in a Taiwanese cohort, suggesting that this factor should be taken into account for early diagnosis [179].

## 7. Clinical Applications of Mycobiome Modulation

Mycobiome composition is usually studied in a disease-focused manner, specifically aiming to describe increased risks of a defined pathology. However, the study of fungal communities may also contribute to the active manipulation of the mycobiome in therapeutic approaches.

One example is the use of fungal-derived immunomodulatory molecules as an attractive or complementary alternative to aggressive antitumor therapies. These strategies consist of the administration of fungal substances, triggering an immune-reactive state in tumor settings [180]. β-glucan administration has been widely studied as an antitumoral therapy, and current knowledge points to macrophage modulation via Dectin-1 interaction as its main mechanism of action [181,182]. Specifically, this molecule can reprogram immunosuppressive M2 macrophages into inflammatory M1 macrophages and thus favor tumor attack. There are numerous clinical trials on the effectiveness of β-glucan administration [182,183], mostly as an adjuvant agent, including neuroblastoma [184], lung cancer [185,186], non-Hodgkin’s lymphoma [187,188], and colorectal cancer [189], among others.

Probiotic supplements are extensively associated with bacterial communities, but fungi also engage in gut homeostasis and can thus be included in these formulations. Specifically, *Saccharomyces cerevisiae* var. *boulardii* supplementation induces therapeutic improvement for inflammatory bowel disease (IBD) [190], diarrhea [191,192] and ulcerative colitis (UC) settings [193,194] by pro-inflammatory cytokine inhibition via NF-κB and ERK/1 pathway impairment, and by directly suppressing bacteria overgrowth and, as previously mentioned, cleavage of *C. difficile* toxins [195].

It is worth mentioning that other *Saccharomyces cerevisiae* strains have been proposed as a probiotic agent for IBD [196], colitis [197], or enterohemorrhagic *E. coli* infections [198]. *S. cerevisiae* has even been studied as a part of a post-biotic preparation, meaning a preparation where *S. cerevisiae* is used to obtain fermentation-derived bioactive compounds, mainly fungal cell wall components (e.g., β-glucans and mannans) [199,200].

Even after sustained research on new fungal-derived probiotics, limited species have shown potential as alternative probiotic agents [201]. Some of these include *D. hansenii* [202,203], *Kluyveromyces marxianus* [204], *Yarrowiali polytica* [205], *Pichia kudriavzevii* [206], *Torulaspora delbrueckii* [207], but their current application still relies on the need for more biosafety studies and regulatory requirements.

The gut mycobiome also holds significant importance in the case of fecal microbiota transplantation (FMT), specifically applied in the treatment of *C. difficile* [208]. Notably, over-representation of *C. albicans* was associated with decreased FMT success and, conversely, patients classified as “responders” showed increased relative abundance of *Saccharomyces* and *Aspergillus*. Interestingly, in UC patients, higher *C. albicans* abundance prior to FMT was associated with responsiveness [209], but a higher presence of the microorganism decreased response in a post-FMT setting. This led to the conclusion that *C. albicans* contributed to a supporting niche suitable for FMT engraftment.

Current knowledge on cancer-associated fungi shows that the mycobiome communities can act as predictive biomarkers [210]. As previously stated, multiple studies have highlighted the diagnostic potential of the mycobiome composition, as is the case with the *Ascomycota/Basidiomycota* ratio for CRC [210] or the *A. sidowii* presence in lung adenocarcinoma [176]. Additionally, the mycobiome composition can also represent a useful tool in the era of precision medicine [211]. For instance, the ASCAs and the perinuclear antineutrophil cytoplasmic antibodies (pANCAs) levels serve as a diagnostic tool to distinguish between Crohn’s disease and UC [212]. The study of the prognostic value of fungi composition can lead to surprising observations, such as the fact that the mycobiome may have a predictive role in abdominal aortic aneurysm (AAA), as it was shown that AAA patients display an increase in *Candida species* and a reduction of *Saccharomyces cerevisiae* [25,213].

The current scientific attention and research directed at immunotherapy, especially at immune checkpoint inhibitors (ICIs) and chimeric antigen receptors (CARs), reinforces the importance of the immune-mycobiome axis, but few studies have focused on the specific role of fungi in immunotherapy. There is limited information that associates the expression of immune checkpoint molecules with the presence of the fungal receptor Dectin-1 [210]. Specifically, dectin-1 upregulates PD-L1 surface expression upon neutrophil exposure to β-glucans [214], and CTLA-4 expression by T_regs_ also increases upon Dectin-1 activation [215]. Other studies have evaluated the effect of mycobiota dysbiosis in ICI blockade therapy, such as Szóstak and colleagues, who associated *Saccharomyces paradoxus* with better responses in anti-PD-1 treatment, whereas high levels of *Malassezia restricta* and *C. albicans* led to worse responses [216]. A multi-kingdom gut microbiota study identified several biomarkers for ICI blockade responses, especially when combining fungi and bacterial data [217]. Interestingly, they identified *Schizosaccharomyces octosporus* as a central hub in responder patients for its short-chain fatty acid production. Beyond this, the role of the gut mycobiome in anti-PD-1 and anti-PD-L1 therapy has been studied in the context of FMT, showing that donors can be stratified into enterotypes based on mycobiome composition, distinguishing a favorable and an unfavorable profile that significantly differ in terms of their immune, metabolic, and bacteriome profiles [218]. Other studies performing a similar approach for hepatocellular carcinoma [219] or including multiple solid tumors [220] also show promising results, suggesting that further research in this field will lead to improved treatments.

## 8. Concluding Remarks

There is a crucial need to further our knowledge on the composition and roles of the human mycobiome in health and disease to enable a complete functional scenario of the bacteriome. Future research should consider eco-systemic approaches, recognizing and highlighting the intricate interactions between the different components of the human microbiome and its host. This implies reaching beyond the fungal component dealt with in this review, to embrace also protists, archaea, and viruses that co-inhabit these ecological niches, in constant interaction, leading to tolerance or response by the immune system and their impact on specific diseases.

A deep understanding of the mycobiome physiology precedes the way to innovative clinical approaches in many disease categories and tailored antifungal therapies. 

## Figures and Tables

**Table 2:** Predominant age-dependent gastrointestinal fungal species.

Age Group	Predominant Fungal Species	Study
10 days to 3 months after birth	*Rhodotorula mucilaginosa* and *Debaryomyces hansenii* (from breast milk or formula)	[41]
>1–2 years	*Ascomycota*, *Zygomycota*, and *Basidiomycota*, with *Candida* species and *Saccharomyces cerevisiae* of *Ascomycota* being the most abundant due to the introduction of solid foods.	[41]
Adults	*Candida* spp., *Saccharomyces*, *and Cladosporium*	[3]
Elders (>65 years)	*Penicillium*, *Candida*, *Aspergillus*, and *Saccharomyces*	[42,43]
